# Novel reversibly switchable wettability of superhydrophobic–superhydrophilic surfaces induced by charge injection and heating

**DOI:** 10.3762/bjnano.10.84

**Published:** 2019-04-10

**Authors:** Xiangdong Ye, Junwen Hou, Dongbao Cai

**Affiliations:** 1 School of Mechanical and Electrical Engineering, Shaanxi Key Laboratory of Nano Materials and Technology, Xi’an University of Architecture and Technology, Xi’an 710055, China

**Keywords:** charge injection, heating, reversible wettability, superhydrophilic, superhydrophobic

## Abstract

Reversibly switching wettability between superhydrophobicity and superhydrophilicity has attracted widespread interest because of its important applications. In this work, we propose a reversible superhydrophobic–superhydrophilic conversion induced by charge injection and heating. Different from the conventional electrowetting phenomenon caused by the accumulation of solid–liquid interfacial charges, we discovered a phenomenon where charge injection and accumulation at the solid surface results in a sharp increase in wettability. The wettability of a sprayed SiO_2_ nanoparticle coating on a glass slide was shown to change from superhydrophobic to superhydrophilic by charge injection and heating, and the superhydrophobicity was restored by heating, verifying a reversible superhydrophobic–superhydrophilic conversion. The influence of voltage, temperature, and time on the coating wettability and its durability under reversible conversion have been studied.

## Introduction

Surfaces that are capable of reversibly switchable wettability have attracted increasing interest, especially those able to switch between superhydrophobicity and superhydrophilicity, and hence, the effects of external stimuli on surface wettability have been explored extensively. For instance, chemical valves and sensors may exhibit an excellent wettability responsiveness to external physical and chemical stimuli [1–3]. Stimuli can be divided into: light [4–11], temperature [12–15], pH [16–18], and electric field [19–25] stimulation.

Zhang et al. [4] reported that superhydrophobic titanium dioxide surfaces become hydrophilic with a contact angle of 0° after 240 min of ultraviolet radiation. Nishimoto et al. [5] developed a method for converting superhydrophobic surfaces into superhydrophilic surfaces after only 10 min of ultraviolet irradiation. Gao et al. [6] prepared 18 alkyltrichlorosilane-modified TiO_2_ films for the reversible switching between superhydrophilicity and superhydrophobicity of a wood surface. Feng et al. [7] deposited thin films of titanium dioxide nanorods on glass substrates by a low-temperature hydrothermal method. The films were stored in the dark for two weeks to yield films with a contact angle of 154 ± 1.3°. Film irradiation with ultraviolet light for 2 h rendered the films superhydrophilic with a contact angle of 0°. Li et al. [8] prepared a superhydrophobic and superhydrophilic zinc oxide film on a bamboo surface. The surface was irradiated by ultraviolet radiation/darkroom for 12 h. A storage period of ten days induced the reversible switching between superhydrophilicity and superhydrophobicity. Yang et al. [9] described a simple method for preparing superhydrophobic films using carbon nanotubes. The method did not require chemical modification of the coating. The reversible switching from superhydrophobicity (contact angle of 155°) to superhydrophilicity (contact angle of 0°) can be achieved by ultraviolet irradiation for 40 min and darkroom storage for 24 h. Su et al. [10] described the superhydrophobic and superhydrophilic conversion of a bismuth coating. Superhydrophobic and superhydrophilic surface wettability can be achieved by modification of the bismuth coating by ultraviolet light irradiation for 50 min and exposure to an ethanol solution that contains stearic acid for 3 min. Gu et al. [11] found a way to control the photoinduction of N-12 alkyl mercaptan. It took only 25 min to change from superhydrophobic (contact angle of about 159 ± 1°) to superhydrophilic (contact angle of 0°), and the reverse process took only 30 s.

Esmeryan et al. [12] revealed collapsed superhydrophobicity and conversion to superhydrophilicity upon thermal annealing of the coating at temperatures above 300 °C. Lai et al. [13] prepared a uniform and stable TiO_2_-based nanoband film by electrophoretic deposition. The transformation of hydrogen titanate to porous TiO_2_ (B) and anatase-type TiO_2_ completed the superhydrophilic–superhydrophobic transition but the process was unidirectional and irreversible. Jiang et al. [14] prepared cotton fabrics by a three-step method that comprised cotton fabrics that were modified by (3-aminopropyl)triethoxysilane (APS) and (1*H*,1*H*,2*H*,2*H*-perfluorodecyl)triethoxysilane (PFDTS) at appropriate ratios. The wettability of the cotton fabric could be changed between superhydrophilic (contact angle of 0°) and superhydrophobic (161.3° contact angle) by controlling the temperature to 25–60 °C. Hou et al. [15] studied the influence of drying temperature on the hydrophobicity of PS/TiO_2_. By increasing the drying temperature from 20 °C to 180 °C, the static contact angle of the coating changed from 0 ± 2° to 158 ± 2°.

Yu et al. [16] prepared a surface with pH responsiveness that was superhydrophobic in acidic and neutral water but superhydrophilic in an ordinary environment. Lei et al. [17] reported pH-responsive switching between superhydrophobicity and superhydrophilicity for high oil–water separation efficiency. Lv et al. [18] achieved a reversible transformation between superhydrophobicity and superhydrophilicity using a folded graphene coating that was prepared by ethanol drying and prewetting.

The wettability of droplets on electrodes coated with an insulator thin film can be changed by applying direct or alternating-current potentials. This phenomenon is termed electrowetting [19]. The equilibrium morphology under electrical wetting conditions is determined by the equilibrium of Maxwell stress and Laplace pressure [20–21]. Verplanck et al. [22] reported the reversible electrical wetting of droplets on superhydrophobic silicon nanowires in air and oil environments. At 150 V, the maximum contact angle could be reduced by 23° by electrical wetting in a reversible manner. Li et al. [23] studied the diffusion of droplets of ionic liquids on an insulating electrode subjected to an external voltage. The catalytic effect of a vertical electric field on the reversible transition of graphene surface water droplets from hydrophobic to hydrophilic was studied at a shutdown voltage [24]. Nbelayim et al. [25] achieved the reversible transformation of graphene from hydrophobic to hydrophilic driven by a direct-current voltage. Cui et al. [26] tested droplets of different sizes using a voltage of 3 V and a 25 V electrical wetting. The maximum contact angle decreased from 150 ± 0.1° to 20° and the contact-angle saturation conditions changed with droplet size. Zahiri et al. [27] reported the reversible active control of surface wettability of copper electrodeposition by an electrochemical process. The surface wettability could be controlled from superhydrophobic to superhydrophilic. When the sample was dried at room temperature or heated at 100 °C, the wettability could be reversed.

Compared with the electrowetting phenomenon caused by electric-field-driven solid–liquid interfacial charges, we discovered a phenomenon where the charge is injected and accumulates on the solid surface driven by a direct-current electric field and results in a sharp increase in wettability. In this study, a SiO_2_ nanoparticle coating sprayed on a glass slide was placed in a high-voltage electric field between two electrodes. The charge was injected through the electrodes and accumulated on the coating by controlling the voltage, temperature, and time. The surface wettability of the coating changed from superhydrophobic to superhydrophilic and was restored by heating.

## Experimental

### Materials and instrumentation

Alcohol (99.7%, AR) and hydrophobic silica (16–25 nm diameter) was provided by Evonik Degussa Co. Ltd. (Germany). The varnish (Voep-1) was from Shijiazhuang Paint Company. All reagents were used as received without further purification. A high-voltage power supply (Tianjin Dongwen) was used for wettability modification of the silica surfaces. An S3000 scanning electron microscope (SEM, Japan Hitachi Group) and a Kelvin probe force microscope (Bruker Dimension Icon, Brook) with a SCM-PIT probe (Pt/Ir coated tips, 2.8 N/m, 75 kHz, Pt/Ir reflective coating) were used for surface-potential characterization. Atomic force microscopy (AFM) was used to characterize the physical morphology (Bruker Dimension Icon, Brook). The water contact angle (CA) of all surfaces was measured using a JC2000C1 contact angle measurements system (Shanghai Zhongchen Digital equipment). At room temperature, 5 µL of deionized water was absorbed by a 5 µL needle tube and dripped vertically on the surface of the sample. The final contact angle of the sample surface was the average value of five points on the sample.

### Coating preparation

0.2 g of SiO_2_ was slowly dispersed in 10 mL of alcohol and sonicated for 30 min. 1 mL varnish was added to a mixed solution of SiO_2_/alcohol and magnetically stirred for 30 min. The final coating solution was sprayed onto glass slides and cured at room temperature for 2 h (spraying distance 20 cm, spraying time 3 s).

### Experiment setup

**Figure 1:**
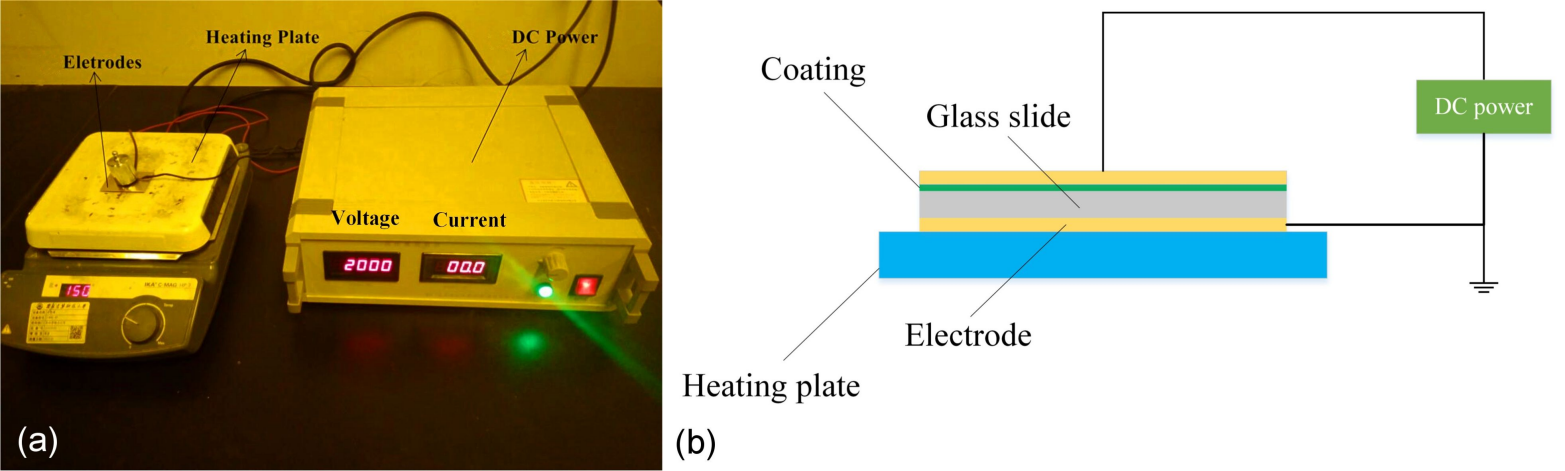
Images of the experimental device for charging.

For studying the effect of the DC electric field on the wettability of the coating, an experiment setup as shown in Figure 1a was used. The device was composed of a power supply, electrodes, a heating plate, and a glass slide with a sprayed coating. The coated glass slide was sandwiched between two Cu electrodes, and half of the slide was treated under an electric field between the electrodes, and the other half was not treated by the electric field, as shown in Figure 1b.

## Results and Discussion

### Coating morphology and wettability

**Figure 2:**
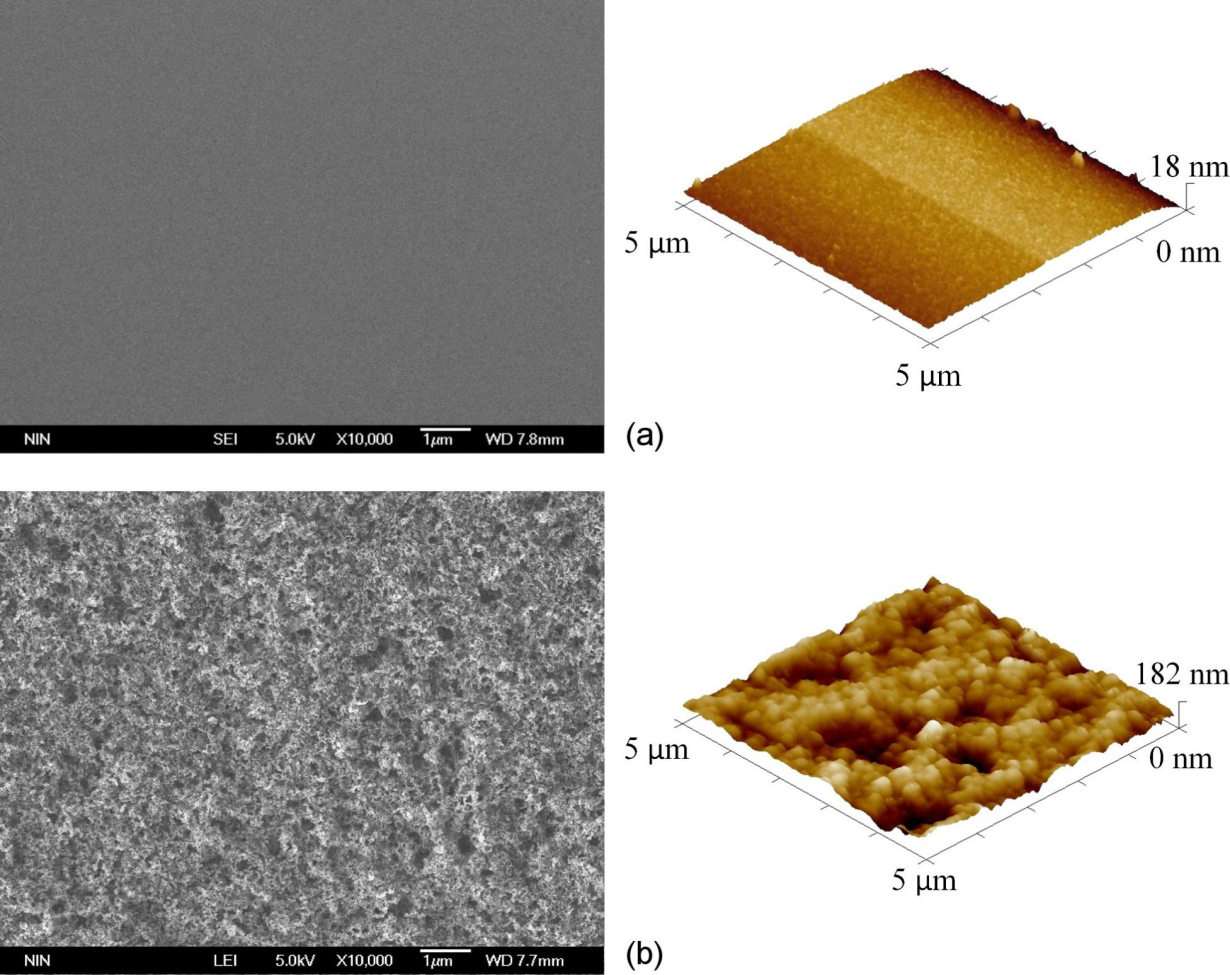
SEM and AFM images of a glass slide a) before and b) after the spray coating procedure.

**Figure 3:**
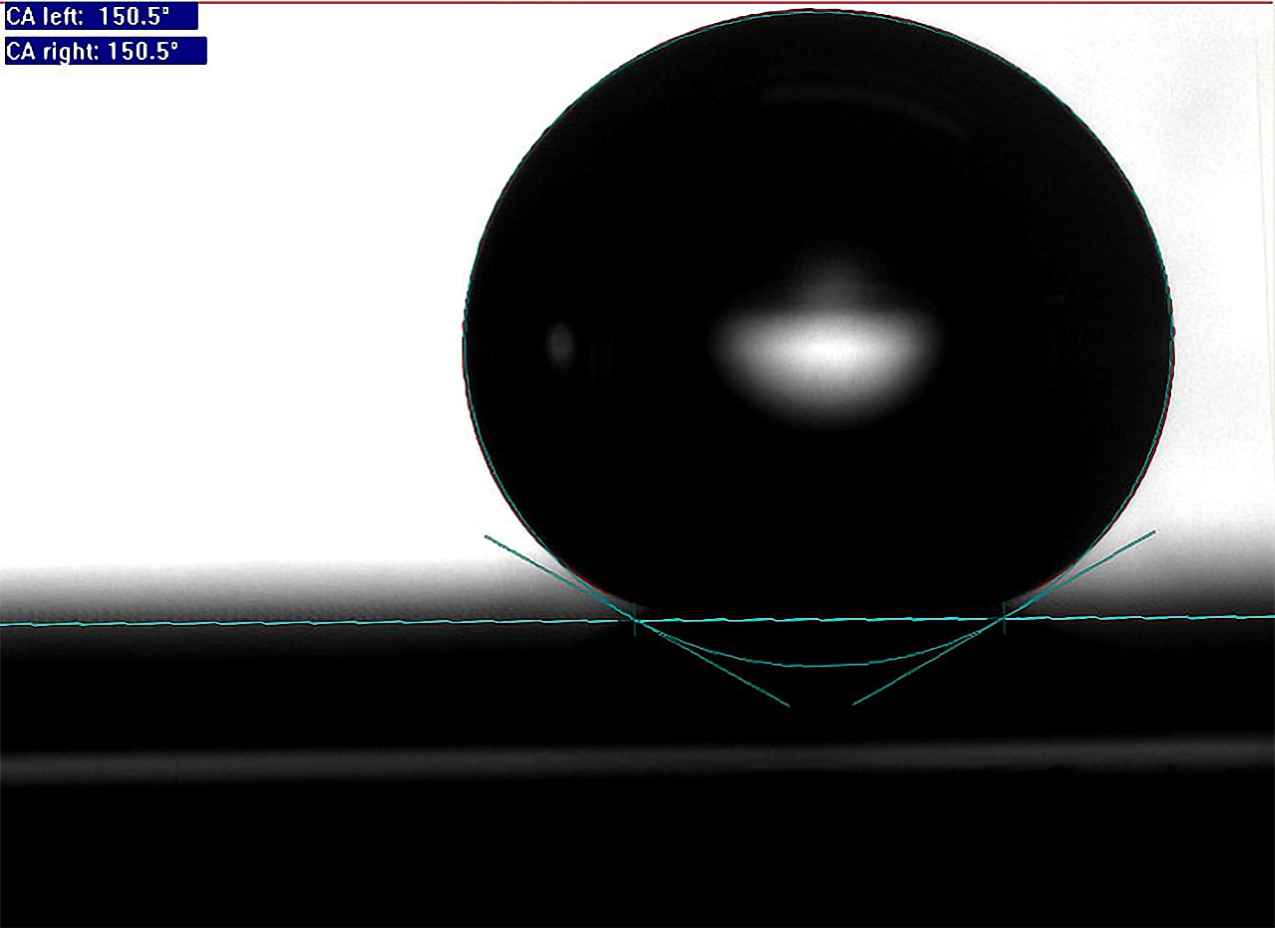
Contact angle of water on the sprayed coating, before charging.

The SEM and AFM images of the glass slide before and after the spray coating procedure are shown in Figure 2a and Figure 2b, respectively. It is obvious that after spraying, the glass-slide surface became rough with a large number of particles that formed irregular micro- and nanoscale composite structures, which increased the roughness of the coating surface and changed the wettability of the coating into superhydrophobic. The surface wettability was inspected and Figure 3 shows that the coating was superhydrophobic with a contact angle of 150.5°. The advancing angle of the layer is 151.2 ± 0.7°, and the receding angle is 150.2 ± 0.3°.

### Change from superhydrophobic to superhydrophilic in an electric field

#### Effect of voltage on coating wettability

**Figure 4:**
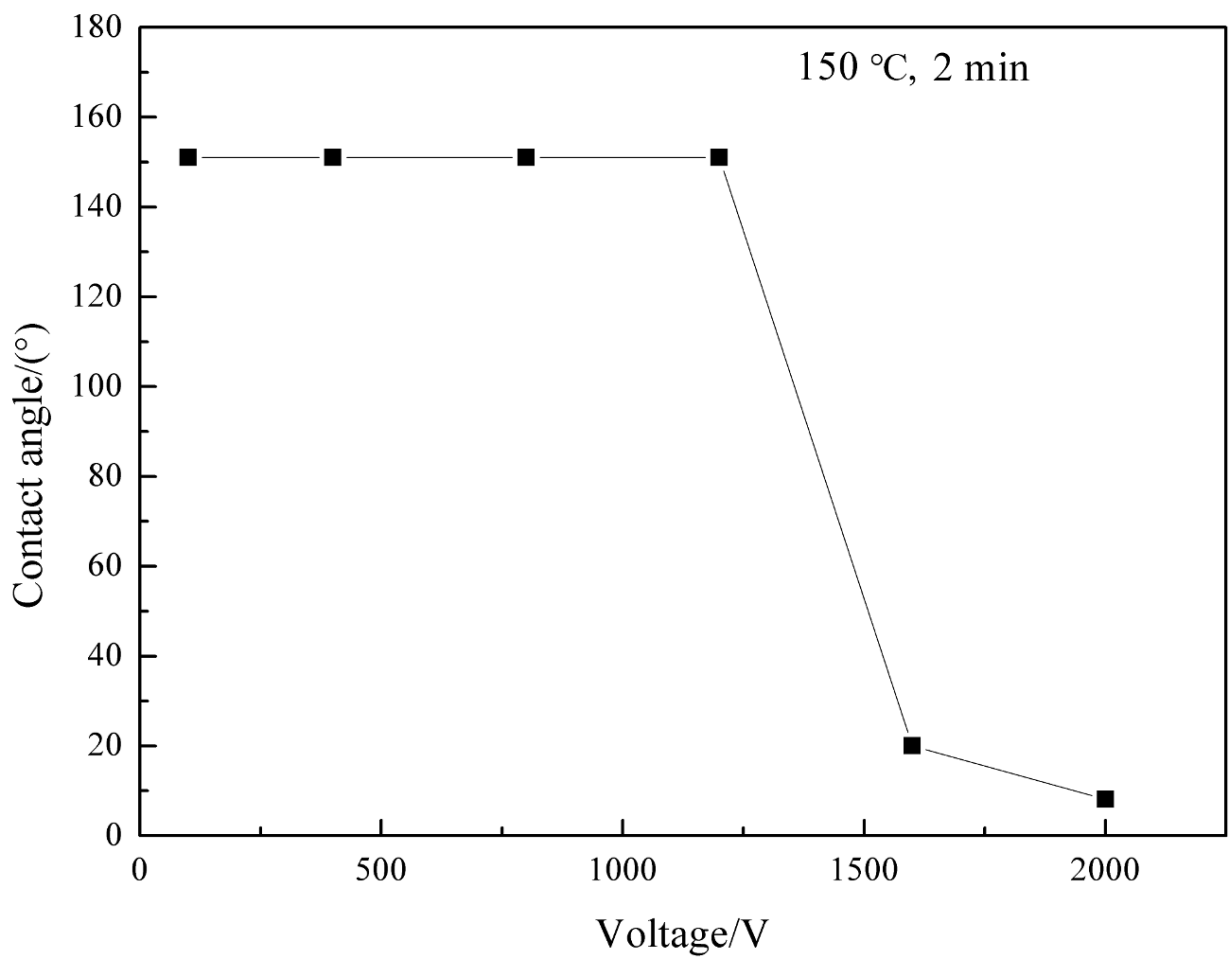
Changes in the contact angle as a function of voltage for the coating (*T* = 150 °C, *t* = 2 min).

**Figure 5:**
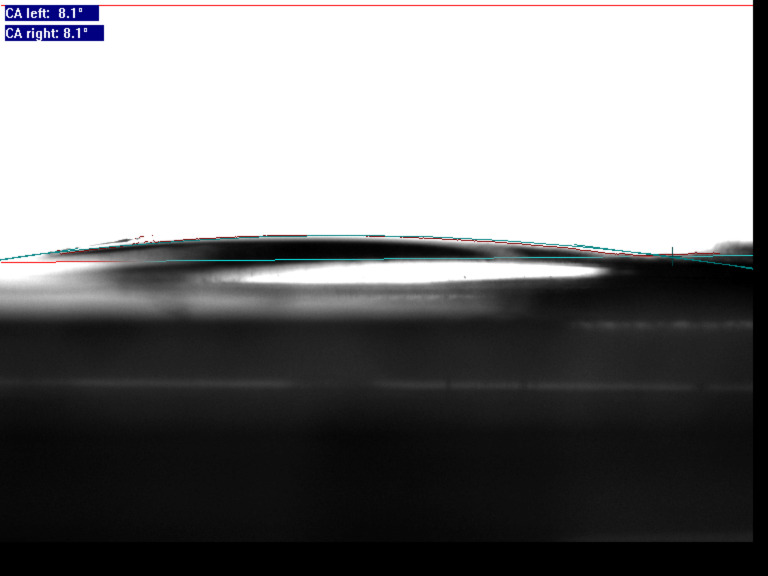
Contact angle of the coating after charging for 2 min at 2000 V.

**Figure 6:**
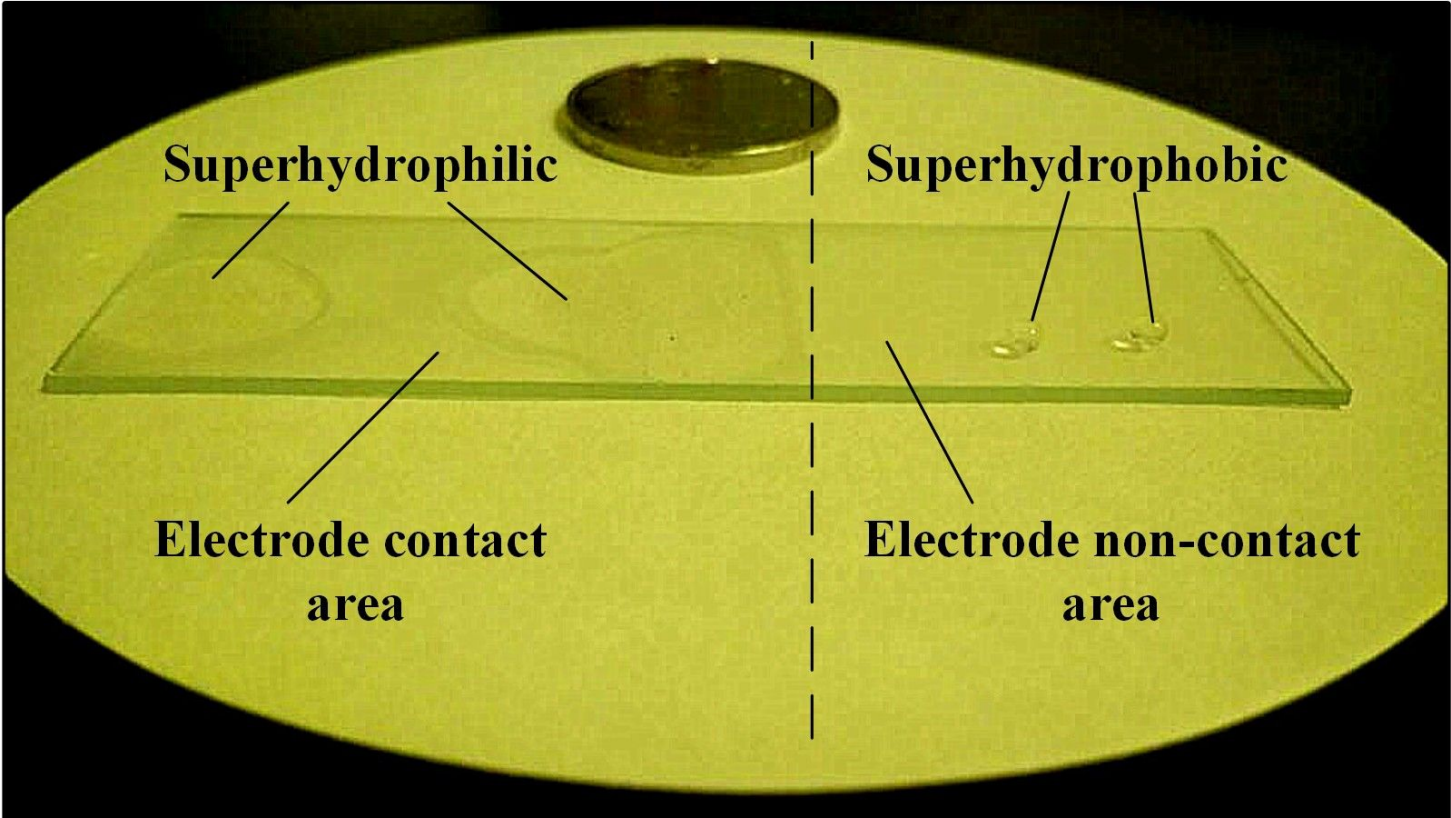
Contrast in wettability of the electrode contact area (after charging for 2 min at 2000 V) and the non-contacted area for the same coating/sample.

To evaluate the effect of voltage on the coating wettability, the voltage was changed gradually while the temperature was kept at 150 °C for 2 min. The results are shown in Figure 4. When the voltage increased from 100 V to 1200 V, the contact angle remained unchanged at 150.5°. As the voltage increased to 1600 V, the contact angle decreased sharply to 20°. When the voltage increased to 2000 V, the contact angle decreased to 8.1° and the coating became superhydrophilic, as shown in Figure 5. The electrode contact area of the coating became superhydrophilic but the uncontacted area remained superhydrophobic, as shown in Figure 6.

**Figure 7:**
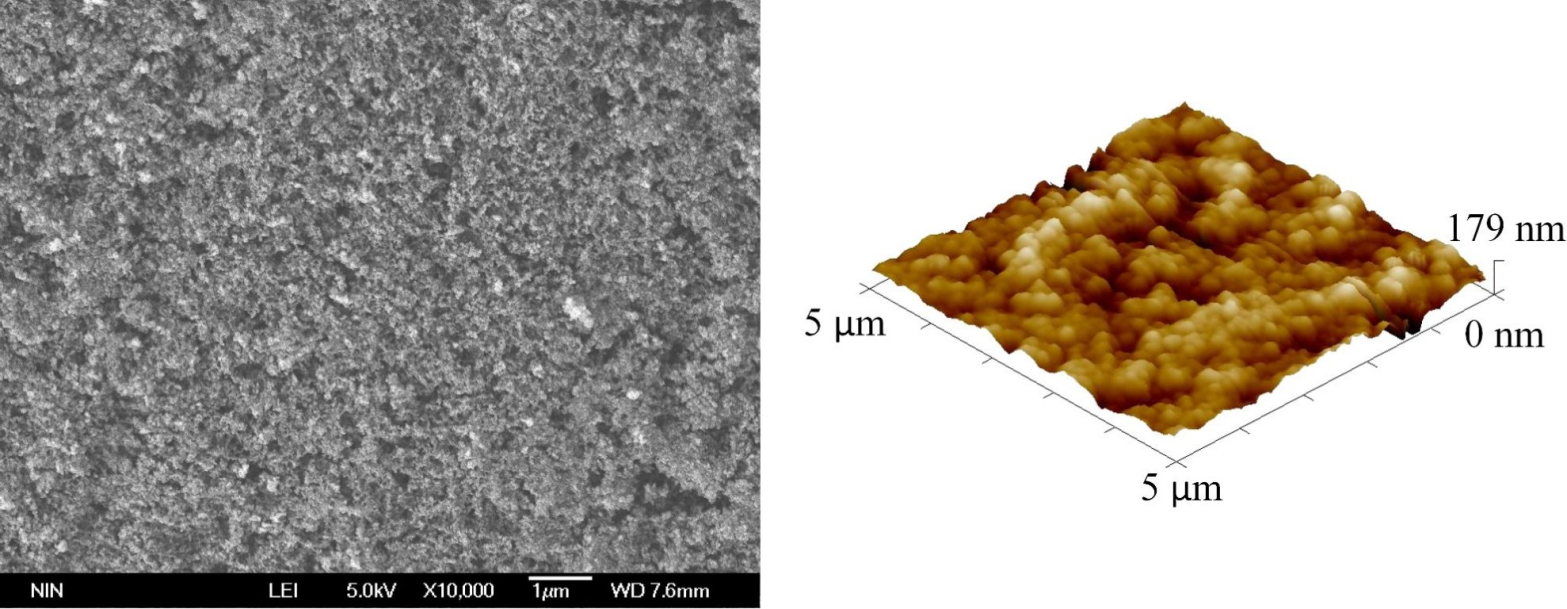
SEM and AFM image of the electrode contact area (after charging for 2 min at 2000 V) in the coating.

We examined the morphology of the electrode contact area in the coating by SEM and AFM, as shown in Figure 7. There was no obvious morphology change in electrode contact area compared with the electrode non-contact area (compared to Figure 2b). Therefore, we conclude that the change in coating wettability was not induced by the surface morphology. To establish the reason for the wettability change, Kelvin probe force microscopy was used to detect the surface potential of the electrode contact area and the electrode non-contact area in the coating. The surface potential was measured at five points in each sample area. The results are shown in Table 1.

**Table 1:** Electrical potential of points within the electrode contact after charging for 2 min at 2000 V and points within the non-contact area.

Position	Electrode contact area/V	Electrode non-contact area/V

point 1	−0.24	0.89
point 2	−0.25	1.29
point 3	−0.24	0.61
point 4	−0.25	0.07
point 5	−0.25	0.22
average value	−0.25	0.61

**Figure 8:**
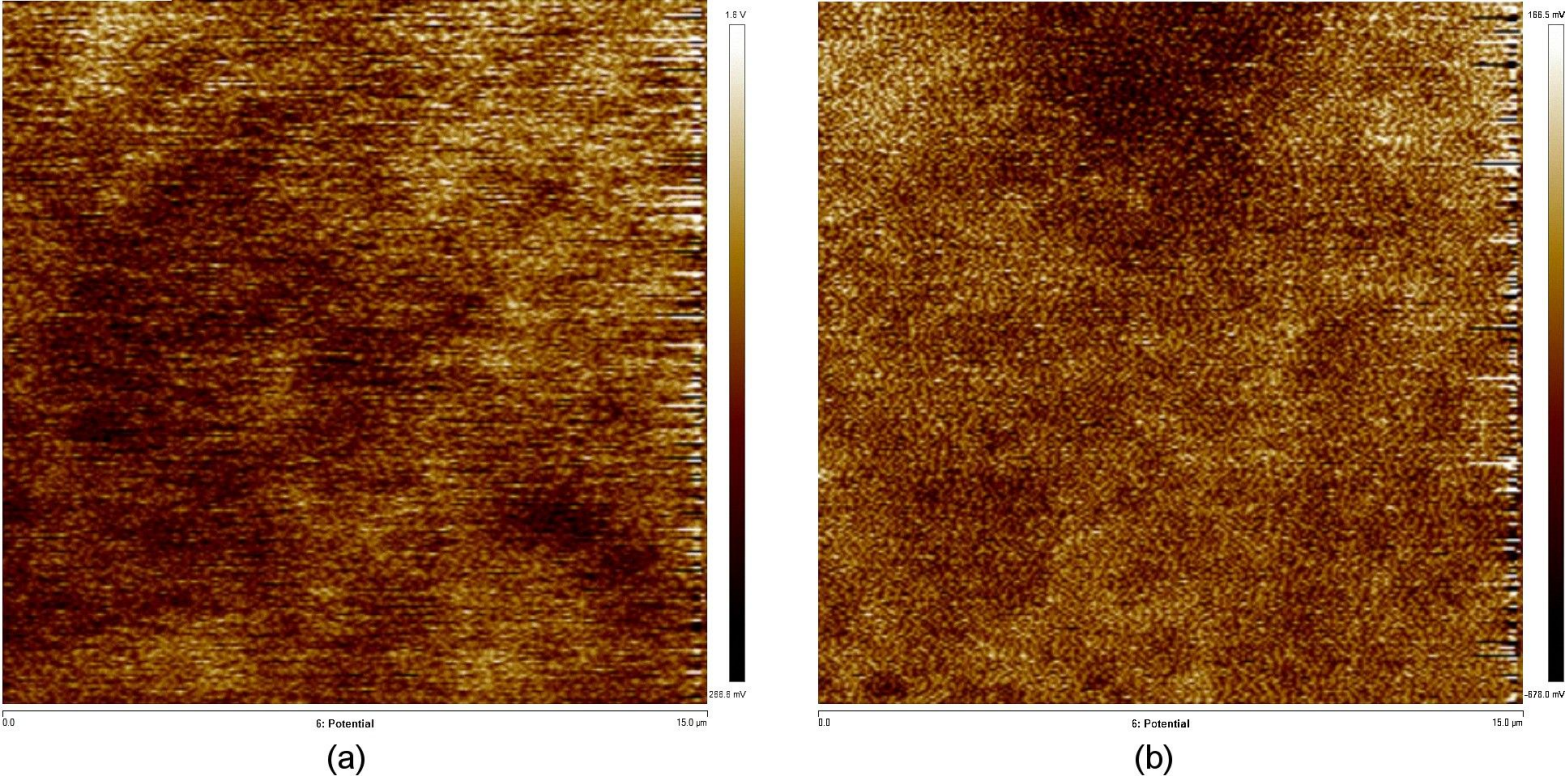
Potential distribution of point 1: a) electrode non-contact area and b) electrode contact area.

Because there are many KPFM images (CPD) for the whole data set in Table 1, as an example, we have only shown the KPFM image for the point 1 to show the contact potential difference, as shown in the Figure 8.

As shown in Table 1 and Figure 8, the surface potential in the electrode non-contact area showed significant fluctuations (0.07–1.29 V). However, the surface potential in the electrode contact area remained almost constant (−0.25 V to −0.24 V). According to the basic principle of Kelvin probe force microscopy, in the uncharged area, the surface potentials fluctuate significantly and result in random data, whereas in the charged area, the surface potentials remain steady. As we have known, the electrowetting phenomenon caused by electric-field-driven solid–liquid interfacial charges will change the wettability from hydrophobic to very hydrophilic [19–27]. We thought that the charges injected and accumulated on the solid surface by a direct-current electric field may also change the surface wettability. For example, the charges that were injected in the electrode contact area converted the coating wettability from superhydrophobic to superhydrophilic. Moreover, only when the voltage is high enough, the charges injected into the solid surface will be enough to change the coating wettability from superhydrophobic to superhydrophilic.

#### Influence of temperature on coating wettability

**Figure 9:**
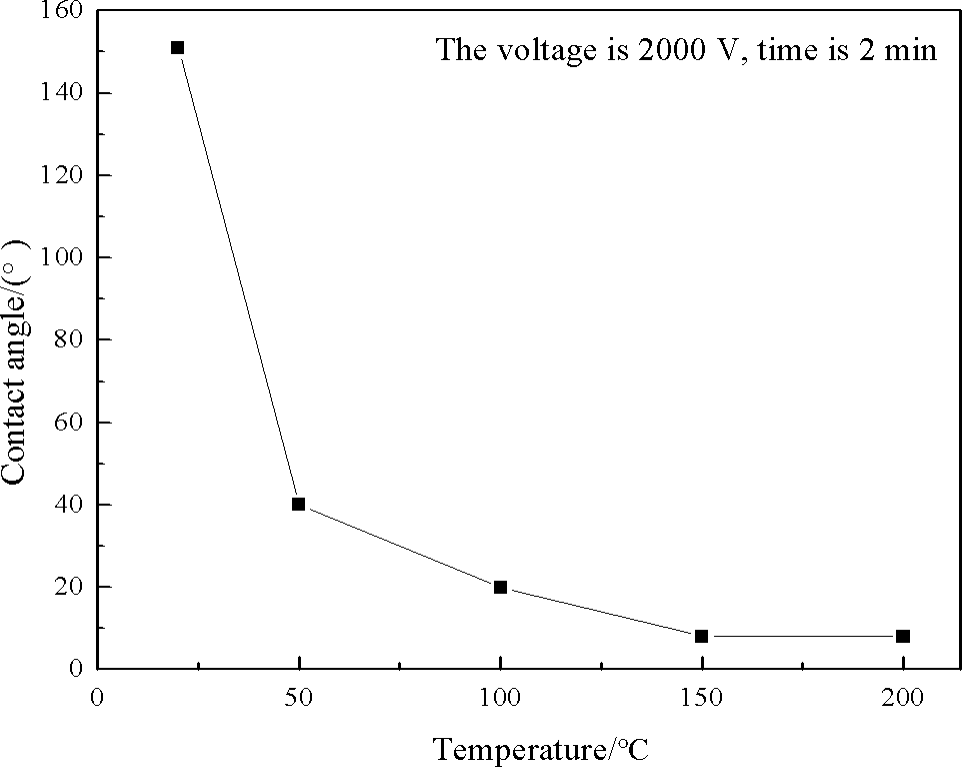
Changes in the contact angle of the coating upon heating (2000 V, 2 min).

To evaluate the effect of heating temperature on the coating wettability, the temperature was changed gradually while the voltage was kept at 2000 V and the charging time was kept at 2 min, and the results are shown in Figure 9. At room temperature, the coating contact angle remained at 150.5°. When the coating was heated at 50 °C, the coating wettability changed from superhydrophobic to weak hydrophilic (from 150.5° to ≈40°). At 100 °C, the contact angle of the coating decreased to ≈20°. As the temperature increased to 150 °C, the coating wettability changed to superhydrophilic. The contact angle remained unchanged when the temperature exceeded 150 °C, and the coating wettability remained superhydrophilic. We speculated that the reason for the enhanced wettability transition from superhydrophobic to superhydrophilic with increasing temperature may be the following: the increase of temperature will promote the thermal motion of the molecules in the solid, which in turn will help the charges inject into the solid surface during the process of electric field application.

#### Effect of charging time on durability of superhydrophilicity

**Figure 10:**
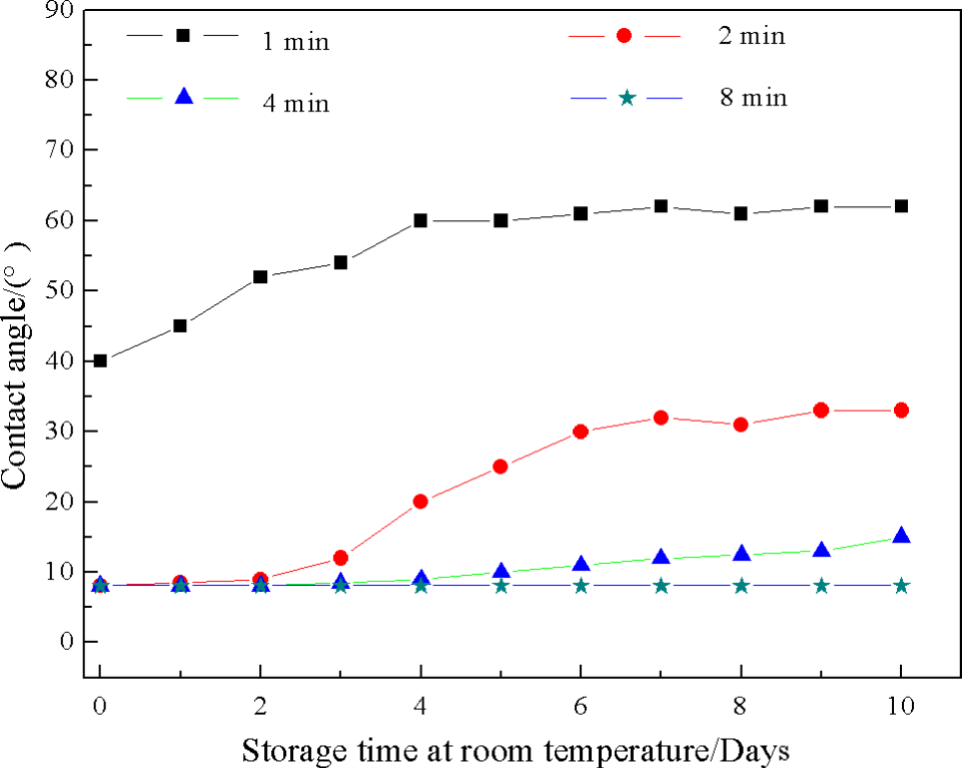
Changes in contact angle of the coating as a function of storage time for four charging times at 2000 V and 150 °C.

When the charging time is too short, the coating wettability cannot change from its original state (superhydrophobic) to superhydrophilic. For example, at 2000 V and 150 °C, the contact angle of the coating changed from 150.5° to 40° after 1 min. Only when the charging time is increased, such as at 2 min, will the coating achieve superhydrophilicity. The durability of the obtained superhydrophilicity of the coating varied with charging time. These results are presented in Figure 10.

Figure 10 shows that the original superhydrophobic coating charged for 2 min became superhydrophilic and the coating remained superhydrophilic after storage for 1 day at room temperature. However, after 2 days and 10 days at room temperature, the contact angle of the coating increased to 9° and 33°, respectively. The contact angle of the coating charged for 4 min also increased gradually at room temperature and the highest contact angle could be restored to 15° after 10 days. After 10 days at room temperature, the surface wettability of the coating charged for 8 min remained superhydrophilic. From the above results, it is obvious that the durability of superhydrophilicity increased with increasing charging time. We speculate the reason is that more charges will be injected into the solid surface when the charging time is longer, which in turn will increase the durability of superhydrophilicity.

#### Recovery from superhydrophilicity to superhydrophobicity with heating

Heating treatment was used to restore the coating wettability from superhydrophilicity to superhydrophobicity. For example, a superhydrophilic coating was obtained by charging at 2000 V and 150 °C for 2 min. The superhydrophilic coating was treated at different heating temperatures and heating times, and the results are shown in Table 2.

**Table 2:** Changes in the contact angle of a superhydrophilic coating (obtained by charging at 2000 V and 150 °C for 2 min) with heating temperature and heating time.

Heating temperature /°C	Heating time	Final contact angle /°

room temperature	10 d	33
100	48 h	90
150	5 h	150.5
200	40 min	150.5
300	10 min	150.5

After storing at room temperature for 10 days, the contact angle of the superhydrophilic coating changed to 33°. At a heating temperature of 100 °C, the contact angle of the superhydrophilic coating increased to 90° after 48 h. At a heating temperature of 150 °C, the contact angle of the superhydrophilic coating increased to 150.5° after 5 h. After heating at 200 °C for 40 min, the superhydrophilic coating recovered its superhydrophobicity (from 8.1° to 150.5°). An increase in heating temperature, for example to 300 °C, results in a shortening of the heating time for the recovery of superhydrophobicity to 10 min.

#### Reversible conversion of coating superhydrophilicity–superhydrophobicity

**Figure 11:**
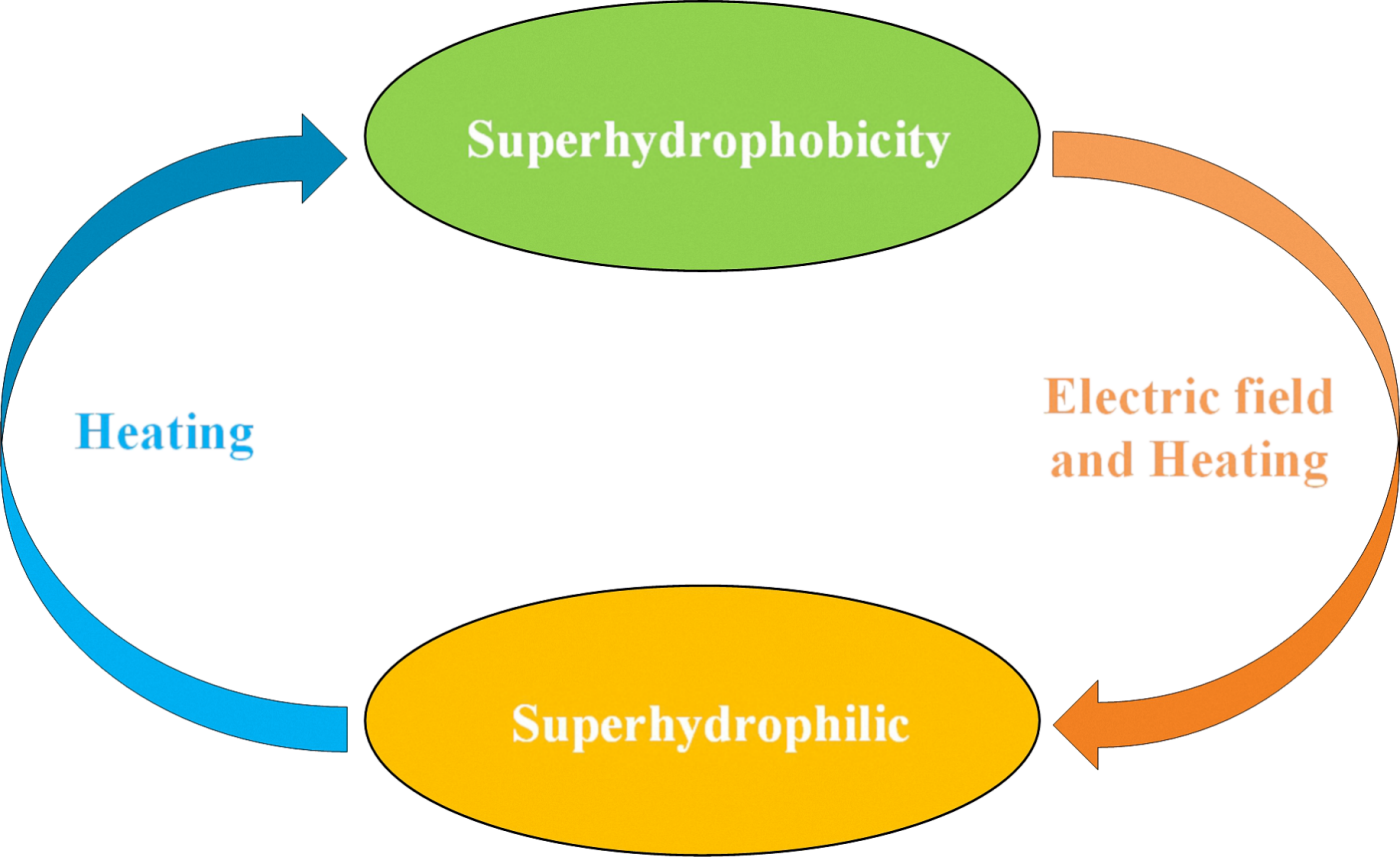
Reversible conversion of coating superhydrophilicity–superhydrophobicity.

The above discussion indicates that the coating can exhibit a reversible superhydrophobic–superhydrophilic conversion. The details are shown schematically in Figure 11. The surface wettability of the coating changed from superhydrophobic to superhydrophilic under the combined action of an electric field and heating. The surface wettability of the coating recovered from superhydrophilic to superhydrophobic with heating.

**Figure 12:**
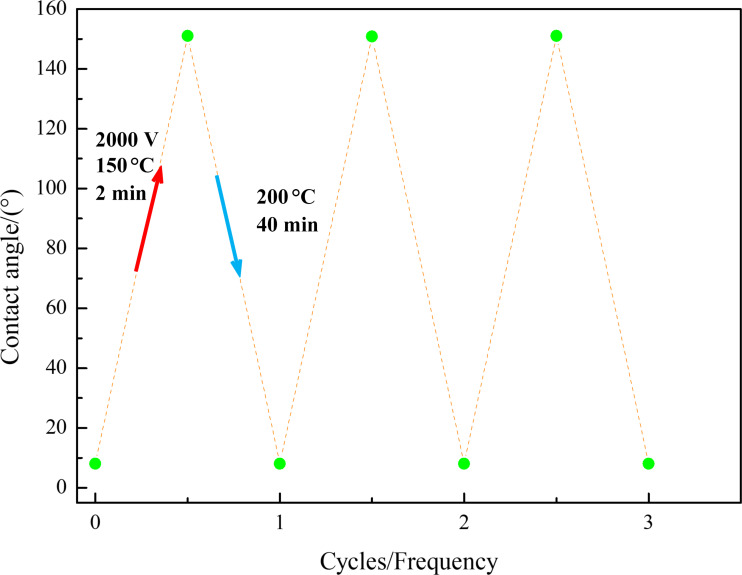
Durability of reversible conversion of coating superhydrophilicity–superhydrophobicity.

To verify the durability of this reversible process, a superhydrophobic coating was charged at 2000 V and 150 °C for 2 min. Then, the obtained superhydrophilic coating was heated at 200 °C for 40 min to restore its superhydrophobicity. The process was repeated three times, as shown in Figure 12.

## Conclusion

The surface wettability of the initially superhydrophobic, SiO_2_ nanoparticle, sprayed coating was transformed to superhydrophilic. This switchable wettability was induced by the combined action of an electric field and heating. The application of sufficient voltage, a higher temperature, and a longer charging time resulted in the charge injected on the coating surface, which converted the wettability of the coating from superhydrophobic to superhydrophilic. The coating was shown to reversibly recover from superhydrophilicity to superhydrophobicity with heating.
